# More Than a Lymph Node in the Neck: A Rare Synovial Cell Sarcoma with Carotid Artery Mass Effect

**DOI:** 10.7759/cureus.5187

**Published:** 2019-07-21

**Authors:** Michael J Yoo, Matthew J Streitz

**Affiliations:** 1 Emergency Medicine, Brooke Army Medical Center, Fort Sam Houston, USA

**Keywords:** neck mass, synovial sarcoma, ultrasound, mass effect, carotid artery, lymph node

## Abstract

The authors discuss a case of a previously healthy 33-year-old man who presented with a subacute neck mass, found to be a rare synovial cell sarcoma with mass effect on the carotid vessels. This report demonstrates the utility of point of care ultrasound and computed tomography (CT) in the workup of the patient’s neck mass. Additionally, we synthesize findings from previous studies that recommend approaching neck masses in adults with a high index of suspicion for malignancy.

## Introduction

Neck masses have a broad differential diagnosis, including lymph nodes, goiter, infection, and cancer. While neck masses with acutely emergent implications are uncommon, all neck masses in adults should raise suspicion for malignancy. Here, we review recommendations from recently published clinical practice guidelines that help clinicians risk-stratify patients. Though these guidelines are not tailored specifically for the emergency department (ED), they nonetheless provide excellent direction for approaching an undifferentiated neck mass in an adult. We present the case of a rare synovial cell sarcoma with mass effect on the carotid vessels that was discovered with the use of bedside ultrasound and computed tomography (CT) of the neck.

## Case presentation

**Figure 1:**
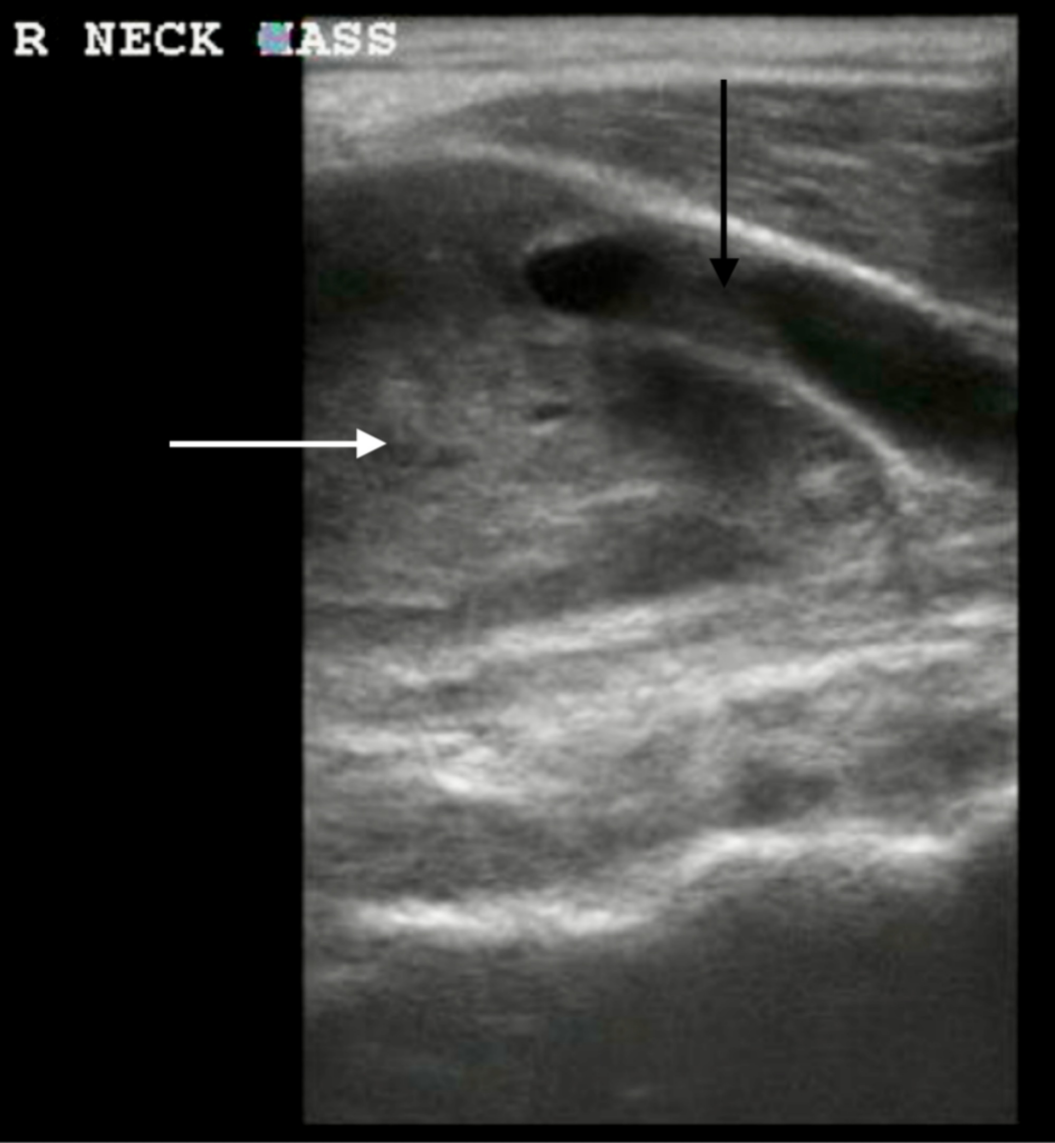
A bedside ultrasound demonstrating a homogenous neck mass (white arrow) at the level of the right carotid artery bifurcation (black arrow).

**Figure 2:**
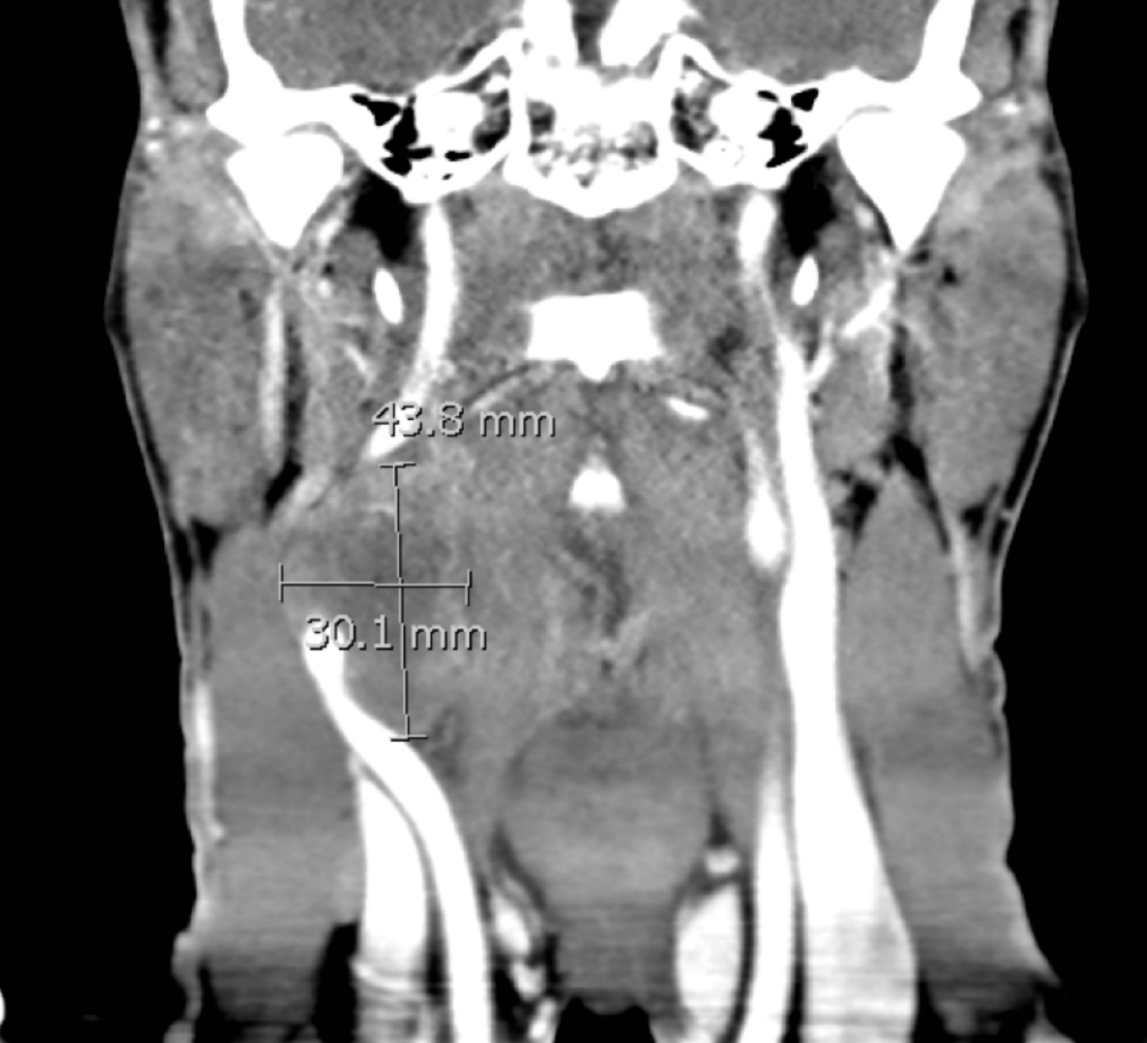
A coronal slice of a contrasted computed tomography scan of the neck, demonstrating a 43.8 mm by 30.1 mm mass at the level of the right common carotid artery bifurcation.

A 33-year-old man presented with increasing right-sided neck swelling over two weeks. The patient endorsed dull pain for five days and chills for three days prior to his presentation to the emergency department (ED). Vitals on arrival were within normal limits. Physical exam revealed a 4 cm nonmobile mass near the right sternocleidomastoid muscle and right tonsillar enlargement but without overlying skin changes or fluctuance. On ultrasound, a neck mass encompassing the right carotid artery was visualized (Figure 1). Further imaging with contrasted computed tomography (CT) of the neck detailed an enhancing mass at the carotid bifurcation, with a lateral mass effect on the common, external, and internal carotid arteries (Figure 2). After consultation with the otolaryngology service and no concerns for acute airway obstruction or hemodynamic instability, the patient was discharged to outpatient surgery, where a 5.1 cm monophasic synovial cell sarcoma was excised at the carotid bifurcation. The patient received adjuvant radiation and six cycles of chemotherapy. Additional imaging revealed metastatic disease to the lungs, and the patient is now on palliative therapy.

## Discussion

Sarcomas are aggressive mesodermal tumors that primarily affect the extremities in the pediatric population and typically present as a growing, painless mass with constitutional symptoms [1]. In adults, sarcomas account for one percent of all malignancies, and sarcomas that affect the head and neck comprise less than 10% of these tumors [2-3]. Furthermore, synovial sarcomas are an even rarer subset of sarcomas that arise from a translocation between chromosomes X and 18 and most commonly present with metastases to the lungs [4-5].^ ^Currently, synovial sarcomas are targeted with wide excision, chemotherapy, and radiation therapy. However, recurrence is common, with only 50% of patients remaining disease-free at 10 years [5].

In 2017, the Otolaryngology-Head and Neck Surgery journal published a clinical practice guideline for approaching neck masses. In addition to withholding antibiotics without suspicion for infection, Pynnonen et al. outlined both historical and physical exam components that suggest an increased risk for malignancy. These include lack of infectious characteristics, lack of fluctuation, duration of two weeks or longer, fixation of the mass, firmness, size greater than 1.5 cm, or overlying ulceration [6]. While these guidelines were not tailored specifically for the emergency department (ED), they provide a reasonable and inclusive set of criteria for ED providers to apply to adults who present with an undifferentiated neck mass.

Several imaging modalities are available in the initial workup of these masses. Though ultrasound is often limited by operator experience, it is both a noninvasive and quick bedside tool that can help clinicians characterize the consistency of the mass, estimate the depth, and observe its effect on surrounding structures. Nevertheless, computed tomography with contrast of the neck is the imaging modality of choice for patients at risk for malignancy [6].

## Conclusions

Neck masses have a broad differential diagnosis and should not always be dismissed as benign lymphadenopathy. In the proper clinical context, neck masses in adults should raise high suspicion for malignancy. Red flags, such as a lack of infectious signs and symptoms, large size, fixation, firmness, and overlying ulceration, warrant further workup. Additionally, bedside ultrasound and computed tomography are excellent imaging modalities in the initial workup of the undifferentiated neck mass.
